# iAIPs: Identifying Anti-Inflammatory Peptides Using Random Forest

**DOI:** 10.3389/fgene.2021.773202

**Published:** 2021-11-30

**Authors:** Dongxu Zhao, Zhixia Teng, Yanjuan Li, Dong Chen

**Affiliations:** ^1^ College of Information and Computer Engineering, Northeast Forestry University, Harbin, China; ^2^ College of Electrical and Information Engineering, Quzhou University, Quzhou, China

**Keywords:** anti-inflammatory peptides, random forest, feature extraction, evolutionary information, evolutionary analysis

## Abstract

Recently, several anti-inflammatory peptides (AIPs) have been found in the process of the inflammatory response, and these peptides have been used to treat some inflammatory and autoimmune diseases. Therefore, identifying AIPs accurately from a given amino acid sequences is critical for the discovery of novel and efficient anti-inflammatory peptide-based therapeutics and the acceleration of their application in therapy. In this paper, a random forest-based model called iAIPs for identifying AIPs is proposed. First, the original samples were encoded with three feature extraction methods, including g-gap dipeptide composition (GDC), dipeptide deviation from the expected mean (DDE), and amino acid composition (AAC). Second, the optimal feature subset is generated by a two-step feature selection method, in which the feature is ranked by the analysis of variance (ANOVA) method, and the optimal feature subset is generated by the incremental feature selection strategy. Finally, the optimal feature subset is inputted into the random forest classifier, and the identification model is constructed. Experiment results showed that iAIPs achieved an AUC value of 0.822 on an independent test dataset, which indicated that our proposed model has better performance than the existing methods. Furthermore, the extraction of features for peptide sequences provides the basis for evolutionary analysis. The study of peptide identification is helpful to understand the diversity of species and analyze the evolutionary history of species.

## 1 Introduction

As a part of the nonspecific immune response, inflammation response usually occurs in response to any type of bodily injury (Ferrero-Miliani et al., 2007). When the inflammatory response occurs in the condition of no obvious infection, or when the response continues despite the resolution of the initial insult, the process may be pathological and leads to chronic inflammation (Patterson et al., 2014). At present, the therapy for inflammatory and autoimmune diseases usually uses nonspecific anti-inflammatory drugs or other immunosuppressants, which may produce some side effects (Tabas and Glass, 2013; Yu et al., 2021). Several endogenous peptides found in the process of inflammatory response have become anti-inflammatory agents and can be used as new therapies for autoimmune diseases and inflammatory disorders (Gonzalez-Rey et al., 2007; Yu et al., 2020a). Compared with small-molecule drugs, the therapy based on peptides has minimal toxicity and high specificity under normal conditions, which is a better choice for inflammatory and autoimmune disorders and has been widely used in treatment (de la Fuente-Núñez et al., 2017; Shang et al., 2021).

Due to the biological importance of AIPs, many biochemical experimental methods have been developed for identifying AIPs. However, these biochemical methods usually need a long experimental cycle and have a high experimental cost. In recent years, machine learning has increasingly become the most popular tool in the field of bioinformatics (Zhao et al., 2017; Liu et al., 2020; Luo et al., 2020; Sun et al., 2020; Zhao et al., 2020; Jin et al., 2021; Wang et al., 2021a). Many researchers have tried to adopt machine learning algorithms to identify AIPs only based on peptide amino acid sequence information. In 2017, Gupta et al. proposed a predictor of AIPs based on the machine learning method. They constructed the combined features and inputted them in the SVM classifier to construct the prediction model (Gupta et al., 2017).

In 2018, Manavalan et al. proposed a novel prediction model called AIPpred. They encoded the original peptide sequence by the dipeptide composition (DPC) feature representation method, and then, they developed a random forest-based model to identify AIPs (Manavalan et al., 2018). AIEpred is a novel prediction model and is proposed by Zhang et al. AIEpred encodes peptide sequences based on three feature representations. Based on various feature representations, it constructed many base classifiers, which are the basis of ensemble classifier (Zhang et al., 2020a).

In this paper, we proposed a novel identification model of AIPs for further improving the identification ability. First, we encoded the samples with multiple features consisting of AAC, DDE, and GDC. It has been proven that multiple features can effectively discriminate positive instances from negative ones in various biological problems. Second, we selected the optimal features based on a feature selection strategy, which has achieved better performance in many biological problems. Finally, we used the random forest classifier to construct an identification model based on the optimal features. The experimental result shows that our proposed method in this paper has better performance than the existing methods.

## 2 Materials and Methods

**FIGURE 1:**
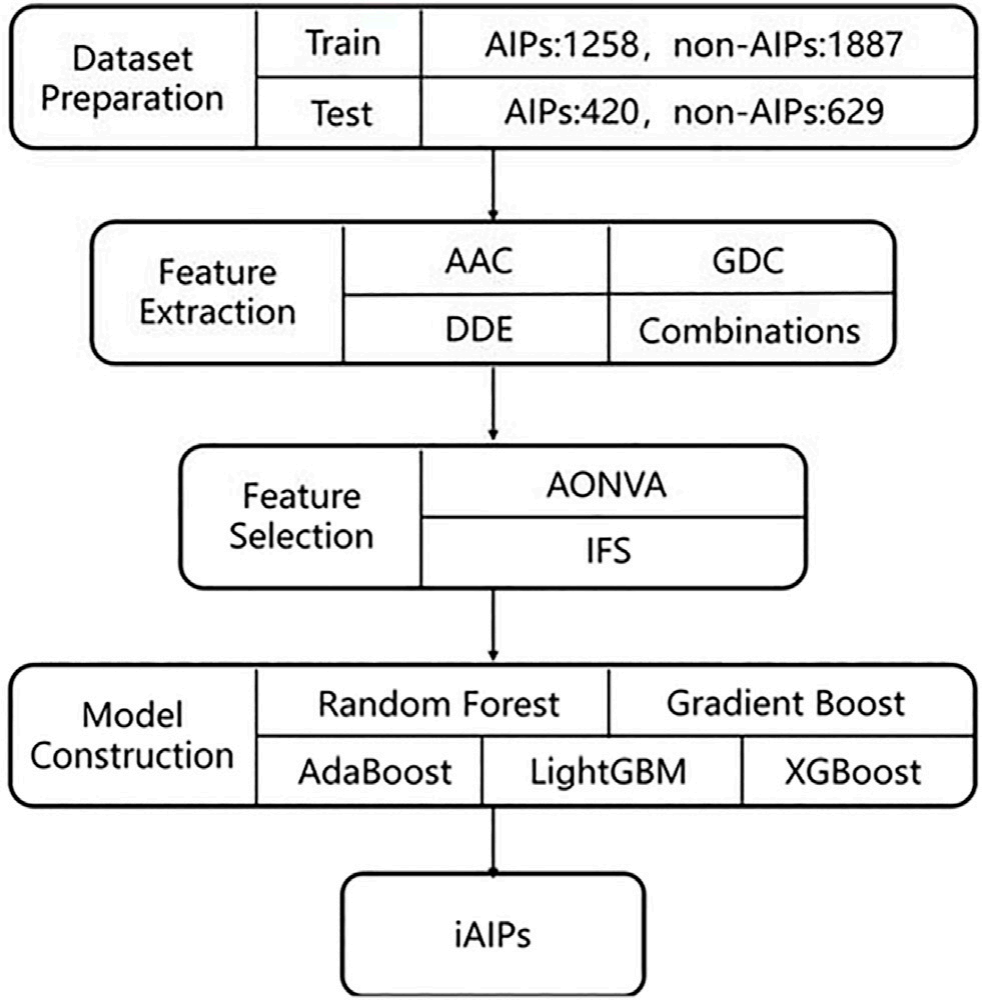
The framework of iAIPs.

Figure 1 gives the general framework of iAIPs proposed in this paper. The framework consists of four steps as follows: 1) Dataset preparation—It collects the data required for the experiment. 2) Feature extraction—It converts the collected sequence data from step 1 into numerical features. 3) Feature selection—removes redundant features from a feature set. 4) Prediction model construction. Each step of the framework will be described as follows.

### 2.1 Dataset Preparation

A high-quality dataset is critical to construct an effective and reliable prediction model. To measure the performance of our model by comparing it with other existing machine learning-based prediction models, we used the dataset with no change proposed in AIPpred (Manavalan et al., 2018). The dataset was first retrieved from the IEDB database (Kim et al., 2012; Vita et al., 2019), and then the samples with sequence identity >80% (Zou et al., 2020) are excluded by using CD-HIT (Huang et al., 2010). The dataset contains 1,678 AIPs and 2,516 non-AIPs. For this dataset, it is randomly selected as the training dataset, which is inputted into the classifier and used to construct the identification model. The training dataset is also used to measure the cross-validation performance of our model. The remaining dataset is used as an independent dataset, which will be used to evaluate the generalization capability of our identification model. In detail, the training dataset consists of 1,258 AIPs and 1,887 non-AIPs, and the independent dataset consists of 420 AIPs and 629 non-AIPs.

### 2.2 Feature Extraction Methods

In the process of peptide identification, finding an effective feature extraction method is the most important step (Liu, 2019; Fu et al., 2020; Cai et al., 2021). In this study, we tried a variety of feature extraction methods and used the random forest classifier to evaluate the performance of those methods. Finally, we chose three efficient feature extraction methods to encode peptide amino acid sequences, including amino acid composition, dipeptide deviation from expected mean, and g-gap dipeptide composition. The details of each feature extraction method are described as follows.

#### 2.2.1 Amino Acid Composition

Different peptide sequences consist of different amino acid sequences. AAC tried to count the composition information of peptides. In detail, AAC calculates the frequency of occurrence of each amino acid type (Wei et al., 2018a; Liu et al., 2019; Ning et al., 2020; Yang et al., 2020; Zhang and Zou, 2020; Wu and Yu, 2021). The computation formula of AAC is as follows:

$$AAC\left(j\right)=\frac{N\left(j\right)}{L},\;\;\;\;\;\;\;j\in \left\{A,C,D,E,F,...,Y\right\}$$

where *L* denotes the length of the peptide, which is the number of characters in the peptide, *AAC* (*j*) denotes the percentage of amino acid j, *N* (*j*) denotes the total number of amino acid *j*. The dimension of AAC is 20.

#### 2.2.2 Dipeptide Deviation From the Expected Mean

According to the dipeptide composition information, DDE computes deviation frequencies from expected mean values (Saravanan and Gautham, 2015). The feature vector extracted by DDE is generated by three parameters: theoretical variance (TV), dipeptide composition (DC), and theoretical mean (TM). The formulas of the three parameters are as follows:

$$D_{C}\left(j\right)=\frac{n_{j}}{L-1}$$

where $n_{j}$ denotes the occurred frequency of dipeptide *j*, and *L* denotes the length of peptide sequences.

$$T_{M}\left(j\right)=\frac{C_{j1}}{C_{N}}\times \frac{C_{j2}}{C_{N}}$$

*C*_*j*1_ denotes the number of codons that encode for the first amino acid, and *C*_*j*2_ denotes the number of codons that encode for the second amino acid in the dipeptide *j*. CN denotes the total number of possible codons.

$$T_{V}\left(j\right)=\frac{T_{M}\left(j\right)\left(1-T_{M}\left(j\right)\right)}{L-1}$$

The formula of DDE(i) is as follows.

$$DDE\left(j\right)=\frac{D_{C}\left(j\right)-T_{M}\left(j\right)}{\sqrt{T_{V}\left(j\right)}}$$

#### 2.2.3 G-Gap Dipeptide Composition

GDC is used to measure the correlation of two non-adjacent residues; its dimension is 400 (Wei et al., 2018b). GDC can be represented as follows:

$$GDC\left(g\right)=\left(f_{1}^{g},f_{2}^{g},...,f_{400}^{g}\right)$$

where $f_{v}^{g}$ is the frequency of v (v = 1,2, …, 400), and it can be calculated as:

$$f_{v}^{g}=\frac{N_{v}^{g}}{\sum_{v=1}^{400}N_{v}^{g}}$$

where $N_{v}^{g}$ denotes the number of the v-th g-gap dipeptide in a given peptide. In this study, every peptide has a different length; the minimum length is 5. Therefore, we set the range of g from 1 to 4. For the different values of g, we represent the feature as GDC-gap1, GDC-gap2, GDC-gap3, and GDC-gap4.

### 2.3 Feature Selection

In the *Feature extraction methods* section, we introduced the feature extraction method used in this paper. However, like other feature representation methods, our feature representation may also produce many noises (Wei et al., 2014; Wang et al., 2020a; Li et al., 2020; Tang et al., 2020; Wang et al., 2021b). Recently, many feature selection methods for eliminating noise has been used to solve many bioinformatics problems (He et al., 2020), such as TATA-binding protein prediction (Zou et al., 2016), DNA 4mc site prediction (Manavalan et al., 2019), antihypertensive peptide prediction (Manayalan et al., 2019), drug-induced hepatotoxicity prediction (Su et al., 2019), and enhance-promoter interaction prediction (Hong et al., 2020; Min et al., 2021).

Likewise, we will use a two-step feature selection method to solve the noise of features. In detail, the feature is first ranked based on the ANOVA score. Then, based on the orderly features, we use the incremental feature selection (IFS) strategy to generate different feature subsets, the feature subset with optimal performance is selected as the optimal feature subset. In the *Result and discussion* section, we will give the experiments about feature extraction, in which we will verify the effectiveness of our feature representation.

#### 2.3.1 Analysis of Variance

In this work, the feature is first ranked based on the ANOVA score. For every feature, ANOVA calculated the ratio of the variance between groups and the variance within groups, which can test the mean difference between groups effectively (Ding et al., 2014). The score is calculated as follows:

$$S\left(t\right)=\frac{S_{B}^{2}\left(t\right)}{S_{W}^{2}\left(t\right)}$$

where *S* (*t*) is the score of the feature t, $S_{B}^{2}\left(t\right)$ is the variance between groups, and $S_{W}^{2}\left(t\right)$ is the variance within groups. The formula of $S_{B}^{2}\left(t\right)$ and $S_{W}^{2}\left(t\right)$ is as follows:

$$S_{B}^{2}\left(t\right)=\frac{1}{K-1}{\sum_{i=1}^{K}m_{i}\left(\frac{\sum_{j=1}^{m_{i}}f_{t}\left(i,j\right)}{m_{i}}-\frac{\sum_{i=1}^{K}\sum_{j=1}^{m_{i}}f_{t}\left(i,j\right)}{\sum_{i=1}^{K}m_{i}}\right)}^{2}$$

$$S_{w}^{2}\left(t\right)=\frac{1}{N-K}{\sum_{i=1}^{K}\sum_{j=1}^{m_{i}}\left(f_{t}\left(i,j\right)-\frac{\sum_{j=1}^{m_{i}}f_{t}\left(i,j\right)}{m_{i}}\right)}^{2}$$

where *K* denotes the number of groups, and *N* denotes the total number of instances; $f_{t}\left(i,j\right)$ denote the value of the *j*-th sample in the *i*-th group of the feature *t*.

#### 2.3.2 Incremental Feature Selection

Based on the orderly features, we use the incremental feature selection strategy to generate different feature subsets; the feature subset with optimal performance is selected as the optimal feature subset. In the incremental feature selection method, the feature set is constructed as empty at first, and then the feature vector is added one by one from the ranked feature set. Meanwhile, the new feature set is inputted into a classifier, and then a prediction model is constructed. We evaluate the performance of the model according to some indicators. Finally, the feature subset with the optimal performance is considered as the optimal feature set.

### 2.4 Machine Learning Methods

In this paper, we utilized various ensemble learning classification algorithms to develop identification models, which contain random forest (Ru et al., 2019; Wang et al., 2020b; Ao et al., 2021), AdaBoost, Gradient Boost Decision Tree (Yu et al., 2020b), LightGBM, and XGBoost. In addition, we also tried some traditional machine learning classification algorithms, such as logistic regression and Naïve Bayes. The description of these methods is as follows.

#### 2.4.1 Random Forest

As one of the most powerful ensemble learning methods, random forest was proposed by Breiman (2001). Due to its effectiveness, random forest has been widely used in bioinformatics areas. Random forest can solve regression and classification tasks. To solve the problem, random forest uses the random feature selection method to construct hundreds or thousands of decision trees (Akbar et al., 2020). By voting on these decision trees, the final identification result is obtained. The random forest algorithm used in this paper is from WEKA (Hall et al., 2008), and all parameters are default.

#### 2.4.2 AdaBoost

The AdaBoost algorithm is an iterative algorithm, which was proposed by Freund (1990). For a benchmark dataset, AdaBoost will train various weak classifiers and combine these weak classifiers by sample weight to construct a stronger final classifier. Among samples, low weights are assigned to easy samples that are classified correctly by the weak learner, while high weights are for the hard or misclassified samples. By constantly adjusting the weight of samples, AdaBoost will focus more on the samples that are classified incorrectly.

#### 2.4.3 Gradient Boost Decision Tree

Similar to AdaBoost, Gradient Boost Decision Tree (GBDT) also combines weak learners to construct a prediction model (Friedman, 2001). Different from AdaBoost, GBDT will constantly adapt to the new model when the weak learners are learned. In detail, based on the negative gradient information of the loss function of the current model, the new weak classifier is trained. The training result is accumulated into the existing model to improve its performance (Basith et al., 2018).

#### 2.4.4 LightGBM and XGBoost

Both LightGBM and XGBoost are improved algorithms based on GBDT. LightGBM is mainly optimized in three aspects. The histogram algorithm is used to convert continuous features into discrete features, the gradient-based one-side sampling (GOSS) method is used to adjust the sample distribution and reduce the numbers of samples, and the exclusive feature bundling (EFB) is used to merge multiple independent features. XGBoost adds the second-order Taylor expansion and regularization term to the loss function.

#### 2.4.5 Naïve Bayes

Naïve Bayes is a probabilistic classification algorithm based on Bayes’ theorem, which assumes that the features are independent of each other. According to this theorem, the probability of a given sample classified into class *k* can be calculated as

$$P\left(C_{k}|X\right)=\;\frac{P\left(C_{k}\right)P\left(X|C_{k}\right)}{P\left(X\right)}$$

where the sample has the expression formula of {X, C}.

#### 2.4.6 Other Machine Learning Methods

Other traditional machine learning methods used for performance comparison include J48, logistic, SMO, and SGD. J48 is a decision tree algorithm provided in Weka, which is implemented based on the C4.5 idea. Logistic is a probability-based classification algorithm. Based on linear regression, Logistic introduces sigmoid function to limit the output value to [0,1] interval. SMO and SGD are optimization algorithms provided in Weka. SMO (sequential minimal optimization) is based on support vector machine (SVM), and SGD is based on linear regression.

### 2.5 Performance Evaluation

To measure the performance of our proposed model, we chose four commonly used measurements: SN, SP, ACC, and MCC (Jiang et al., 2013; Wei et al., 2017a; Ding et al., 2019; Shen et al., 2019; Huang et al., 2020). These measurements are calculated as follows.

$$SN=\frac{TP}{TP+FN}$$

$$SP=\frac{TN}{TN+FP}$$

$$ACC=\frac{TP+TN}{TP+TN+FP+FN}$$

$$MCC=\frac{\left(TP\times TN\right)-\left(FP\times FN\right)}{\sqrt{\left(TP+FP\right)\times \left(TP+FN\right)\times \left(TN+FP\right)\times \left(TN+FN\right)}}$$

where *FP*, *FN*, *TN*, and *TP* show the number of false-positive, false-negative, true-negative, and true-positive, respectively. These are widely used in bioinformatics studies, such as protein fold recognition (Shao et al., 2021), DNA-binding protein prediction (Wei et al., 2017b), protein–protein interaction prediction (Wei et al., 2017c), and drug–target interaction identification (Ding et al., 2020; Ding and JijunGuo, 2020).

Furthermore, we also used the receiver operating characteristic (ROC) curve (Hanley and McNeil, 1982; Fushing and Turnbull, 1996) to evaluate the performance of our proposed model. ROC computes the true-positive rate and low false-positive rate by setting various possible thresholds (Gribskov and Robinson, 1996). The area under the ROC curve (AUC) also shows the performance of the proposed model, which is more accurate in the aspect of evaluating the performance of the prediction model constructed by an imbalanced dataset.

## 3 Results and Discussion

To verify the effectiveness of our proposed model, we will measure the performance of our model from different perspectives. The detailed process of these experiments is presented as follows.

### 3.1 Performance of Different Features

In this study, we use a variety of feature extraction methods and their combinations to encode peptide sequences. At first, we measure the effectiveness of single features. The comparison results of the fivefold cross-validation on the training dataset are shown in Table 1.

**TABLE 1:** Performance comparison of various single features.

Feature	SN	SP	ACC	MCC	AUC
Amino acid composition (AAC)	0.529	0.845	0.719	0.398	0.760
Dipeptide deviation for the expected mean (DDE)	0.589	0.854	0.748	0.464	0.784
G-gap dipeptide composition (GDC)-gap1	0.456	0.862	0.700	0.353	0.764
GDC-gap2	0.466	0.852	0.697	0.348	0.751
GDC-gap3	0.454	0.869	0.703	0.361	0.741
GDC-gap4	0.449	0.853	0.692	0.335	0.733
CKSAAGP	0.477	0.861	0.707	0.371	0.732
CTriad	0.215	0.897	0.624	0.155	0.668
GAAC	0.533	0.750	0.663	0.288	0.679
GDPC	0.525	0.826	0.706	0.370	0.727
GTPC	0.470	0.855	0.701	0.357	0.742
TPC	0.304	0.910	0.668	0.277	0.739

Table 1 shows that DDE is much better than other features according to the indicators of AUC, MCC, ACC, SP, and SN. In detail, the AUC value reaches 0.784, which is 2%–11.6% higher than other features. Based on the indicator of AUC, the features of DDE, GDC-gap1, and AAC have the best performance.

To achieve better performance, we further test the performance of multiple features on the basis of DDE, GDC, and AAC. In detail, the GDC feature adopts four different parameters, that is, gap1, gap2, gap3, and gap4. The corresponding feature is GDC-gap1, GDC-gap2, GDC-gap3, and GDC-gap4. The performance comparison of the fivefold cross-validation on the training dataset is shown in Table 2.

**TABLE 2:** Performance comparison of various combined features of fivefold cross-validation on the training dataset.

Feature	SN	SP	ACC	MCC	AUC
AAC+DDE	0.582	0.857	0.747	0.461	0.784
AAC+GDC-gap1	0.483	0.870	0.715	0.388	0.770
AAC+GDC-gap2	0.453	0.871	0.704	0.363	0.773
AAC+GDC-gap3	0.435	0.866	0.694	0.339	0.759
AAC+GDC-gap4	0.447	0.873	0.703	0.360	0.760
DDE+GDC-gap1	0.586	0.858	0.749	0.466	0.790
DDE+GDC-gap2	0.588	0.854	0.748	0.464	0.791
DDE+GDC-gap3	0.583	0.860	0.749	0.466	0.785
DDE+GDC-gap4	0.587	0.851	0.746	0.459	0.784
AAC+DDE+GDC-gap1	0.585	0.860	0.750	0.468	0.794
AAC+DDE+GDC-gap2	0.584	0.852	0.745	0.457	0.790
AAC+DDE+GDC-gap3	0.593	0.857	0.751	0.471	0.784
AAC+DDE+GDC-gap4	0.587	0.855	0.748	0.464	0.785

According to Table 2, the multiple features of AAC + DDE + GDC-gap1 has the best performance. Its value of SN, SP, ACC, MCC, and AUC are 0.585, 0.860, 0.750, 0.468, and 0.794, respectively.

To verify the performance of these combined features, we tested them on the independent test set. Table 3 shows the experimental results on the independent dataset. The results show that the combined features of AAC + DDE + GDC-gap1 have the best performance on the independent dataset.

**TABLE 3:** Performance comparison of various combined features on the independent dataset.

Feature	SN	SP	ACC	MCC	AUC
AAC+DDE	0.564	0.860	0.742	0.450	0.808
AAC+GDC-gap1	0.488	0.884	0.725	0.413	0.799
AAC+GDC-gap2	0.455	0.878	0.708	0.373	0.787
AAC+GDC-gap3	0.448	0.881	0.707	0.371	0.795
AAC+GDC-gap4	0.462	0.865	0.704	0.362	0.783
DDE+GDC-gap1	0.569	0.857	0.742	0.450	0.812
DDE+GDC-gap2	0.560	0.854	0.736	0.437	0.805
DDE+GDC-gap3	0.576	0.857	0.745	0.456	0.808
DDE+GDC-gap4	0.569	0.857	0.742	0.450	0.801
AAC+DDE+GDC-gap1	0.56	0.859	0.739	0.443	0.806
AAC+DDE+GDC-gap2	0.557	0.855	0.736	0.437	0.805
AAC+DDE+GDC-gap3	0.552	0.855	0.734	0.433	0.806
AAC+DDE+GDC-gap4	0.567	0.859	0.742	0.450	0.801

### 3.2 Performance of Different Classifiers

In this study, we chose the random forest algorithm to construct the classifier. To verify the effectiveness of the random forest classifier, we compared its performance with other classifiers. We chose several ensemble classifiers that are similar to the random forest classifier, including AdaBoost, GBDT, LightGBM, and XGBoost. In addition, we also chose some machine learning classifiers, including J48, Logistic, SMO, SGD, and Naïve Bayes.

Based on the best feature combination, which is obtained from previous experiments, we constructed different identification models using different classifiers. The performance of these classifiers on the training dataset is shown in Table 4.

**TABLE 4:** Performance of various classifiers utilizing AAC-DDE-GDC-gap1 feature and fivefold cross-validation on the training dataset.

Classifier	SN	SP	ACC	MCC	AUC
Random forest	0.585	0.860	0.750	0.468	0.794
AdaBoost	0.579	0.743	0.678	0.324	0.661
Gradient Boost Decision Tree (GBDT)	0.583	0.788	0.706	0.379	0.686
LightGBM	0.564	0.754	0.678	0.321	0.659
XGBoost	0.576	0.757	0.684	0.336	0.666
J48	0.552	0.737	0.663	0.292	0.647
Logistic	0.497	0.677	0.605	0.175	0.624
Sequential minimal optimization (SMO)	0.476	0.725	0.626	0.206	0.601
SGD	0.491	0.689	0.610	0.182	0.590
Naïve Bayes	0.483	0.684	0.603	0.168	0.604

The results in Table 4 show that the performance of the random forest classifier is the best, and its AUC value is 10.8%–20.4% higher than other classifiers. To further compare the generalization ability of these classifiers, we test those models on the independent dataset. Table 5 shows the experimental results. The results showed that the random forest classifier is also better than other classifiers on the independent dataset.

**TABLE 5:** Performance of various classifiers based on AAC-DDE-GDC-gap1 feature on the independent dataset.

Classifier	SN	SP	ACC	MCC	AUC
Random forest	0.560	0.859	0.739	0.443	0.806
AdaBoost	0.607	0.809	0.728	0.426	0.708
GBDT	0.640	0.798	0.735	0.443	0.719
LightGBM	0.538	0.859	0.730	0.424	0.698
XGBoost	0.579	0.847	0.740	0.446	0.713
J48	0.524	0.738	0.652	0.266	0.621
Logistic	0.498	0.658	0.594	0.156	0.615
SMO	0.442	0.701	0.598	0.147	0.572
SGD	0.493	0.679	0.604	0.173	0.586
Naïve Bayes	0.486	0.676	0.600	0.162	0.602

### 3.3 The Analysis of Feature Selection

In the extracted features, some feature vectors may be noisy or redundant. To further improve the identification performance, we try to find optimal features by feature selection methods in this section. In this paper, the two-step feature selection strategy is used as the feature selection strategy to eliminate noise. In detail, we first used the ANOVA method to rank feature vectors, and then we used the IFS strategy to filter the optimal feature set.

**FIGURE 2:**
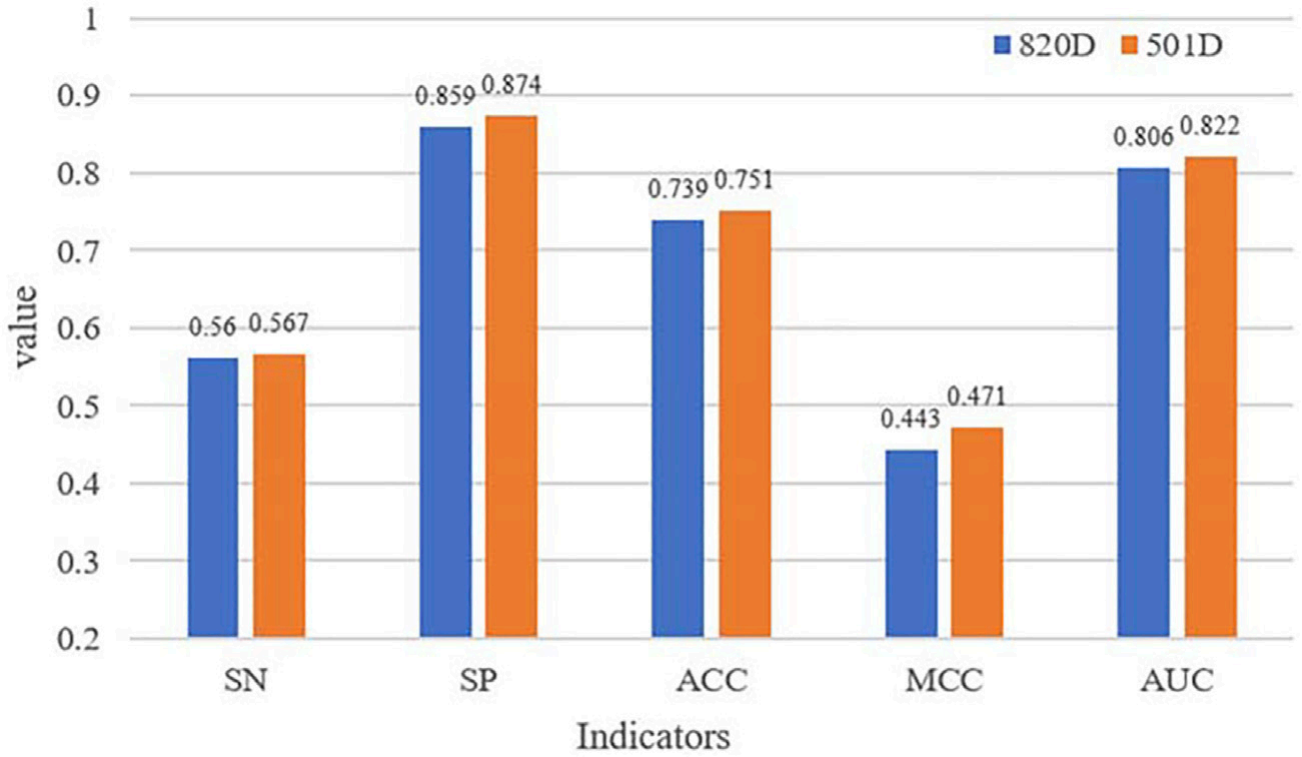
Comparison of identification performance before and after dimensionality reduction.

The comparison of performance before and after dimensionality reduction is shown in Figure 2. All indicators of the selected features have higher values than the original ones. The results suggest that the optimal feature set can improve the overall performance of our identification model and our fewer selected features can still accurately describe AIPs.

### 3.4 Comparison With Existing Methods

Independent dataset test plays an important role in testing the generalization ability of the identification model. Therefore, the independent dataset was used to measure our identification model; the performance of our identification model was compared with existing methods, which contains AntiInflam (Ferrero-Miliani et al., 2007), AIPpred, and AIEpred. Table 6 shows the detailed results of the different methods for identifying AIPs, where the results are ranked according to AUC.

**TABLE 6:** Performance of different identification models on the independent dataset.

Method	SN	SP	ACC	MCC	AUC
AntiInflam (LA)	0.258	0.892	0.638	0.197	0.647
AntiInflam (MA)	0.786	0.417	0.565	0.210	0.706
AIEpred	0.555	0.899	0.762	0.495	0.767
AIPpred	0.741	0.746	0.744	0.479	0.813
iAIPs (our work)	0.567	0.874	0.751	0.471	0.822

As shown in Table 6, the value of our proposed identification model iAIPs in SN, SP, ACC, AUC, and MCC are 0.567, 0.874, 0.751, 0.822, and 0.471, respectively. Furthermore, the same independent dataset-based experimental results showed that the ACC of iAIPs was 0.007–0.186 higher than that of AntiInflam and AIPpred, which is similar to AIEpred. Moreover, according to AUC, our performance is better than the other methods, which is 0.009–0.175 higher than the others. The results indicate that our method has better performance than other existing prediction models.

## 4 Conclusion

In this paper, an identifying AIP model based on peptide sequence is proposed. We tried various features and their combinations, utilized various commonly used ensemble learning classification algorithms and the two-step feature selection strategy. After trying a large number of experiments, we finally constructed an effective AIP prediction model. By conducting a large number of experiments on the training dataset and independent dataset, we verified that our proposed prediction model iAIPs could efficiently identify AIPs from the newly synthesized and discovered peptide sequences, which is better than the existing AIP prediction models.

In the future, the optimization of the feature representation method is a research direction. Especially, the research on a new feature representation method that can adaptively encode peptide sequences is of great significance. Furthermore, other optimization methods and computational intelligence models will be considered for identifying anti-inflammatory peptides. Deep learning (Lv et al., 2019; Zeng et al., 2020a; Zeng et al., 2020b; Zhang et al., 2020b; Du et al., 2020; Pang and Liu, 2020), unsupervised learning (Zeng et al., 2020c), and ensemble learning (Sultana et al., 2020; Zhong et al., 2020; Li et al., 2021; Niu et al., 2021; Shao and Liu, 2021) will be employed when the dataset is large enough.

## Data Availability

Publicly available datasets were analyzed in this study. These data can be found here: http://www.thegleelab.org/AIPpred/.
